# MBNL2 Regulates DNA Damage Response via Stabilizing p21

**DOI:** 10.3390/ijms22020783

**Published:** 2021-01-14

**Authors:** Jin Cai, Ningchao Wang, Guanglan Lin, Haowei Zhang, Weidong Xie, Yaou Zhang, Naihan Xu

**Affiliations:** 1 State Key Laboratory of Chemical Oncogenomics, Tsinghua Shenzhen International Graduate School, Tsinghua University, Shenzhen 518055, China; j-cai17@tsinghua.org.cn (J.C.); 15071441626@163.com (N.W.); 18760068311@163.com (G.L.); zhwyjm@163.com (H.Z.); xiewd@sz.tsinghua.edu.cn (W.X.); zhangyo@sz.tsinghua.edu.cn (Y.Z.); 2 Open FIESTA Center, Tsinghua Shenzhen International Graduate School, Tsinghua University, Shenzhen 518055, China; 3 Institute of Biopharmaceutical and Health Engineering, Tsinghua Shenzhen International Graduate School, Tsinghua University, Shenzhen 518055, China

**Keywords:** MBNL2, RNA-binding protein, p21, DNA damage response, Chk1, cancer

## Abstract

RNA-binding proteins are frequently dysregulated in human cancer and able to modulate tumor cell proliferation as well as tumor metastasis through post-transcriptional regulation on target genes. Abnormal DNA damage response and repair mechanism are closely related to genome instability and cell transformation. Here, we explore the function of the RNA-binding protein muscleblind-like splicing regulator 2 (MBNL2) on tumor cell proliferation and DNA damage response. Transcriptome and gene expression analysis show that the PI3K/AKT pathway is enriched in MBNL2-depleted cells, and the expression of cyclin-dependent kinase inhibitor 1A (p21^CDKN1A^) is significantly affected after MBNL2 depletion. MBNL2 modulates the mRNA and protein levels of p21, which is independent of its canonical transcription factor p53. Moreover, depletion of MBNL2 increases the phosphorylation levels of checkpoint kinase 1 (Chk1) serine 345 (S345) and DNA damage response, and the effect of MBNL2 on DNA damage response is p21-dependent. MBNL2 would further alter tumor cell fate after DNA damage, MBNL2 knockdown inhibiting DNA damage repair and DNA damage-induced senescence, but promoting DNA damage-induced apoptosis.

## 1. Introduction

The cyclin-dependent kinase inhibitor p21^CDKN1A^, also known as p21^Waf1/Cip1^, has multiple functions in cell cycle, apoptosis, DNA replication/repair and transcriptional regulation in response to a variety of stimuli, including ionizing, UV radiation and genotoxic stress [1,2]. The tumor suppressor p53 acting as the main transcriptional factor of p21, various stresses upregulating p53 activity will subsequently induce p21 expression. G1/S cell cycle progression is prevented by p21 primarily through its inhibition on CDK2 activity, which is indispensable for the phosphorylation of Rb and the consequent activation of E2F-dependent gene expression [3,4]. Thus, attributed to the role of cyclin-dependent kinase inhibitor, p21 promotes cell cycle arrest after DNA damage through transcriptional repression on a set of cell cycle-regulatory genes [1].

The expression of p21 is tightly regulated at both transcriptional and post-transcriptional levels. RNA-binding proteins play a crucial role in post-transcriptional regulation of pre-mRNAs, involved in capping, splicing, polyadenylation, export, translation, stability, and other mRNA maturation processes. It has been reported that many RNA-binding proteins post-transcriptionally regulate p21 mRNA stability. For instance, RNPC1, Rbm24, HuR, and HuD bind to the AU-rich elements (AREs) within the 3′ untranslated region (UTR) of p21 transcript and increase p21 mRNA stability [5,6,7,8]. In contrast, PCBP1, PCBP2, PCBP4, and hnRNP K bind to the CU-rich elements in the 3′UTR of p21 mRNA and negatively regulate its stability [9,10,11]. FXR1P muscle-specific isoforms directly interact with p21 mRNA through a conserved G-quadruplex located in its 3′UTR and subsequently reduce its half-life [12]. 

Muscleblind-like (MBNL) proteins are a family of deeply conserved RNA-binding proteins that regulate developmentally programmed alternative splicing [13,14]. Both MBNL and CUGBP Elav-like family (CELF) proteins play predominant roles in the pathogenesis of myotonic dystrophy (DM), in which they are sequestered away from their normal RNA targets, leading to splicing mutations. There are three MBNL paralogs in mammals, MBNL1, MBNL2 and MBNL3, containing highly conserved zinc finger (ZnF) domains which bind preferentially to specific pre-mRNAs to regulate alternative splicing [15,16]. MBNL1 and MBNL2 are broadly expressed across many tissues, whereas MBNL3 primarily exists in the placenta [14]. Except their well-studied functions in DM, recent research has indicated MBNL proteins also actively participate in tumorigenesis and tumor progression through post-transcriptional mechanisms. MBNL1 inhibits tumor metastasis by modulating the stability of DBNL, TACC1, and Snail mRNA [17,18]. MBNL3 activates alternative splicing of lncRNA-PXN-AS1, which increases the expression of PXN to inhibit apoptosis of hepatocellular carcinoma cells [19]. MBNL2 is reported to inhibit tumor growth and invasion in hepatocellular carcinoma [20]; however, the molecular mechanism beyond the anti-tumor effect of MBNL2 is mainly unknown. 

In this report, we performed transcriptome analysis in control and MBNL2 siRNA-transfected cells. Gene expression profiling and KEGG pathway enrichment analysis show that the PI3K/AKT pathway is enriched in MBNL2-depleted cells and p21 is among the most differentially expressed genes. MBNL2 modulates the mRNA and protein levels of p21 independent of p53 but through binding to and post-transcriptionally regulating the stability of p21 mRNA. MBNL2 also affects DNA damage response through controlling the phosphorylation of Chk1. MBNL2 knockdown increases Chk1 S345 phosphorylation and the effect can be rescued by concurrent overexpression of p21. Moreover, MBNL2 alters tumor cell fate upon DNA damage, depletion of MBNL2 inhibiting DNA damage repair and DNA damage-induced cellular senescence, but provoking DNA damage-induced apoptosis.

## 2. Results

### 2.1. MBNL2 Modulates the PI3K/AKT Signaling Pathway

**Figure 1:**
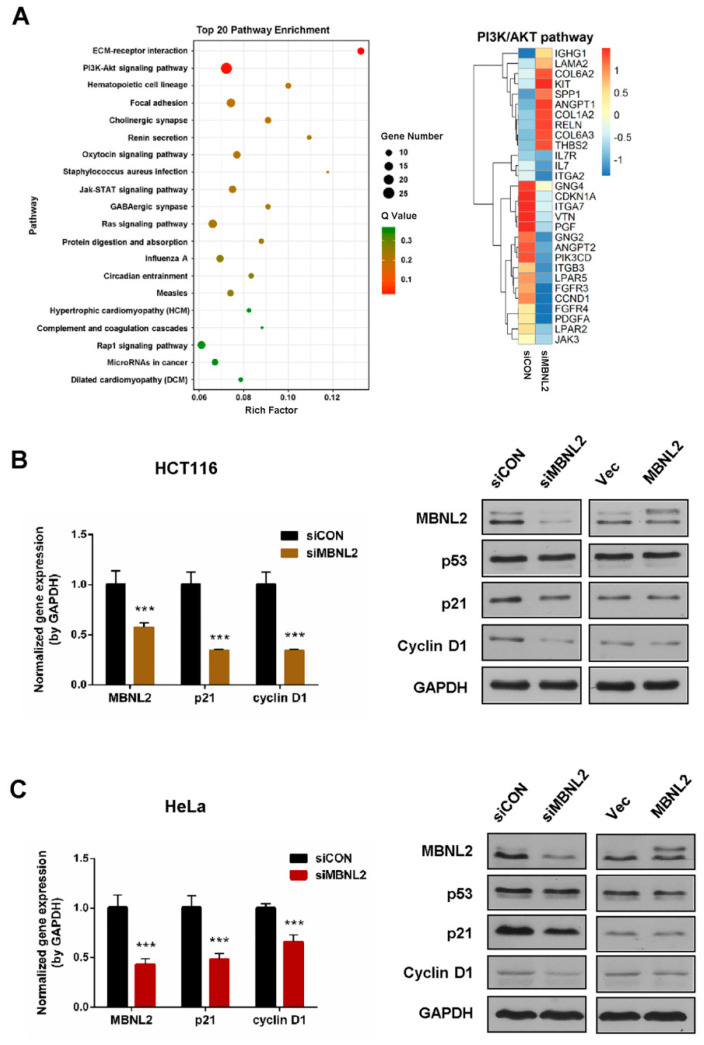
MBNL2 regulates p21 expression. (**A**) KEGG pathway enrichment analysis of RNA-seq data in the HCT116 cells transfected with control or MBNL2 siRNAs (left). The Rich factor refers to the ratio of the number of the differentially expressed genes to the total number of annotated genes. Normalized gene enrichment score of gene sets in the PI3K/AKT pathway was specially analyzed in detail (right). (**B**) HCT116 cells were transfected with control or MBNL2 siRNA, empty vector, or MBNL2-myc plasmids and subjected to qRT-PCR and immunoblotting analysis. (**C**) qRT-PCR and immunoblotting analysis in HeLa cells transfected with control or MBNL2 siRNA, empty vector, or MBNL2-myc plasmids. Abbreviations: si, siRNA; CON, control; Vec, vector. *** *p* < 0.001.

To study the function of MBNL2 on tumorigenesis and tumor progression, we performed transcriptome and gene expression analysis in the HCT116 cells transfected with control or MBNL2 siRNA. The differentially expressed genes were subjected to KEGG pathway enrichment analysis, the PI3K/AKT signaling pathway was significantly enriched in MBNL2-depleted cells (Figure 1A). The PI3K/AKT pathway is an intracellular signal transduction pathway that promotes cell survival and proliferation in response to various extracellular signals [21,22]. CDKN1A (p21), a predominant cell cycle inhibitor, is one of the most differentially expressed genes after MBNL2 depletion in the PI3K/AKT pathway. 

We then performed qRT-PCR and immunoblotting to verify the regulation of MBNL2 on p21 expression. qRT-PCR analysis confirmed that the p21 transcript was significantly reduced in MBNL2-depleted HCT116 cells. Immunoblotting showed that the p21 protein level was also markedly reduced after MBNL2 knockdown, whereas ectopic expression of MBNL2 had no obvious effect on p21 protein expression (Figure 1B). MBNL2 exhibited the same regulatory effect in HeLa cells, both qRT-PCR and immunoblotting demonstrating that p21 expression was largely decreased in MBNL2-depleted HeLa cells (Figure 1C). Moreover, we found that CCND1 (cyclin D1), another important cell cycle regulator in the PI3K/AKT pathway, was also differentially expressed after MBNL2 depletion (Figure 1A) and verified by molecular experiments in both HCT116 (Figure 1B) and HeLa cells (Figure 1C).

### 2.2. MBNL2 Regulates p21 Expression in a p53-Independent Manner 

**Figure 2:**
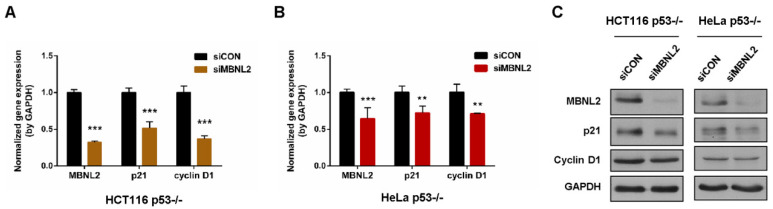
MBNL2 regulates p21 expression independently of p53. (**A**) The mRNA levels of MBNL2, p21, and cyclin D1 were determined in the HCT116 p53−/− or (**B**) HeLa p53−/− cells transfected with control or MBNL2 siRNAs. (**C**) Immunoblotting of MBNL2, p21, and cyclin D1 in the HCT116 p53−/− or HeLa p53−/− cells transfected with control or MBNL2 siRNAs. ** *p* < 0.01, *** *p* < 0.001.

The cyclin-dependent kinase inhibitor p21 is one of the most crucial transcriptional targets of p53 to induce cell cycle arrest, apoptosis, and cellular senescence [1,2]. To determine whether MBNL2 regulates p21 expression through its canonical transcription factor p53, we knocked down MBNL2 in p53−/− HCT116 or HeLa cells. In accordance with the decrease in wide type cells, p21 mRNA level was significantly reduced upon depletion of MBNL2 in p53−/− cells (Figure 2A,B). Similarly, the p21 protein level was markedly reduced in the cells transfected with MBNL2 siRNA in p53-null cells (Figure 2C). These results indicate the regulatory effect of MBNL2 on p21 expression is p53-independent.

### 2.3. MBNL2 Regulates p21 mRNA Stability and Tumor Cell Proliferation

**Figure 3:**
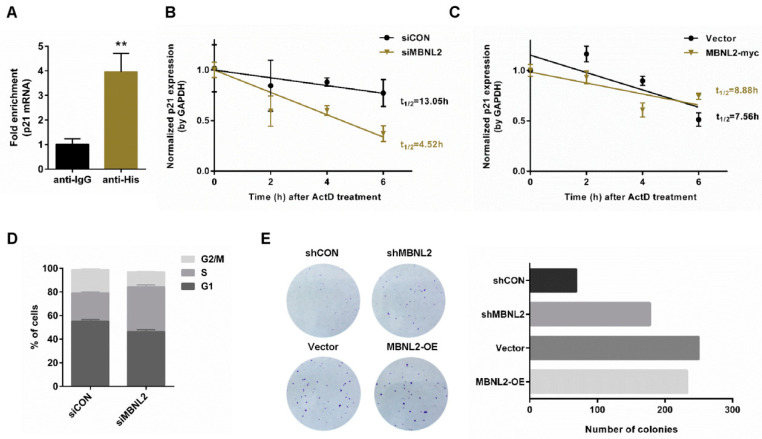
Depletion of MBNL2 destabilizes p21 mRNA and promotes tumor cell proliferation. (**A**) HCT116 cells transfected with His-MBNL2 plasmids were subjected to RIP, and MBNL2-associated p21 mRNA was determined by qRT-PCR. (**B**) HCT116 cells transfected with control or MBNL2 siRNA, (**C**) empty vector, or MBNL2-myc plasmids were treated with 5 mg/mL ActD for the indicated time. Normalized expression (qRT-PCR) of the remaining p21 mRNA after ActD treatment was performed using linear regression analysis. (**D**) Flow cytometry analysis of the percentage for G1, S, and G2/M cells in the HCT116 cells transfected with the indicated siRNAs, pulse-labeled with 1 mg/mL BrdU for 30 min before harvesting, and subjected to PI/anti-BrdU staining. (**E**) Representative images (left) and quantification (right) of the colony formation assay in the HCT116 cells infected with the indicated lentiviral constructs and left for 8-day culture. ** *p* < 0.01.

A significant post-transcriptional function of RNA-binding proteins is to modulate mRNA stability. To study whether RNA-binding protein MBNL2 is involved in regulation of p21 mRNA stability, we performed RIP and qRT-PCR analysis. HCT116 cells were transfected with His-tagged MBNL2 plasmids and immunoprecipitated with the anti-His antibody. MBNL2-associated RNAs were purified for cDNA synthesis and quantified by qRT-PCR with specific p21 primers. Interestingly, we found that p21 mRNA was co-immunoprecipitated with the His-MBNL2 protein, indicating MBNL2 could physically associate with p21 mRNA (Figure 3A). Then, we overexpressed or knocked down MBNL2 in HCT116 cells, treated cells with the transcription inhibitor actinomycin D (ActD), and detected the half-life of the existing p21 mRNA. Depletion of MBNL2 accelerated p21 mRNA decay, whereas ectopic expression of MBNL2 slightly delayed p21 mRNA decay, proving MBNL2 could regulate the stability of the p21 transcript (Figure 3B,C). 

G1/S transition is prevented by p21 due to its function of the cyclin-dependent kinase inhibitor, hence, depletion of p21 can promote cell cycle progression and cell proliferation [1,2]; we then explore the role of MBNL2 in tumor cell proliferation. HCT116 cells were transfected with control or MBNL2 siRNA and subjected to PI/BrdU flow cytometry to detect cell cycle distribution. Depletion of MBNL2 increased the percentage of S phase cells (Figure 3D). We also performed colony formation assay; depletion of MBNL2 significantly increased the number of colonies whereas ectopic expression of MBNL2 only mildly reduced cell proliferation (Figure 3E). These results indicate that MBNL2 modulates cell cycle progression and tumor cell proliferation through post-transcriptional regulation on p21.

### 2.4. MBNL2 Regulates Chk1 Activation and DNA Damage Response

**Figure 4:**
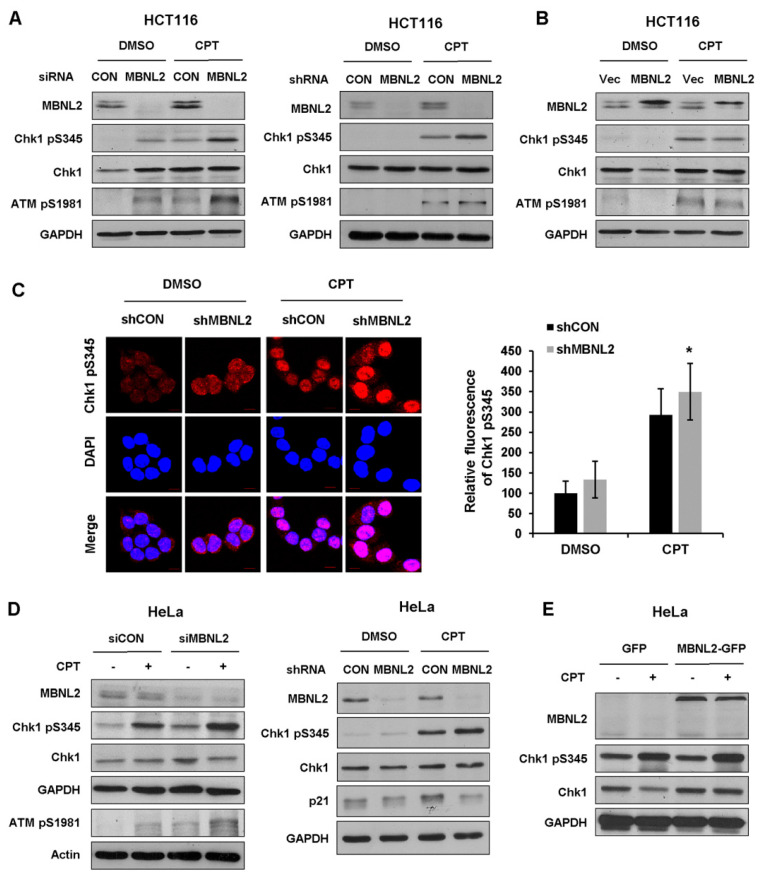
Depletion of MBNL2 upregulates DNA damage response. (**A**) HCT116 cells transfected with control or MBNL2 siRNA (left) and shRNA lentiviral constructs (right), (**B**) empty vector, or His-MBNL2 plasmids were treated with 1 μM CPT for 4 h or mock-treated, then subjected to immunoblotting analysis. (**C**) Representative images (left) of Chk1 pS345 immunostaining in the HCT116 cells transfected with control or MBNL2 shRNA lentiviral constructs and treated with 1 μM CPT for 4 h or mock-treated. Relative fluorescence intensity was quantified (right). (**D**) Immunoblotting of Chk1 pS345 in the HeLa cells transfected with control or MBNL2 siRNA (left) and shRNA lentiviral constructs (right), (**E**) empty vector, or MBNL2-GFP plasmids and treated with 1 μM CPT for 4 h or mock-treated. * *p* < 0.05.

Functional deficiency of DNA damage response and repair mechanism is closely associated with genome instability and cell transformation. Several studies indicate RNA-binding proteins also take part in the regulation of DNA damage response, then affect tumorigenesis and tumor progression. We next explore functions of the RNA-binding protein MBNL2 in DNA damage response. HCT116 cells were transfected with control or MBNL2 siRNA/shRNA and then treated with DNA damage reagent, the topoisomerase I inhibitor camptothecin (CPT) for 4 h to induce DNA damage response. Phosphorylation of Chk1 by ATM/ATR at S345 within the C-terminal regulatory domain is in particular essential for both the DNA damage and replication checkpoint responses in vertebrates. Immunoblotting revealed that the phosphorylation levels of checkpoint kinases Chk1 S345 and ATM S1981 were strongly increased after MBNL2 depletion upon DNA damage (Figure 4A). On the contrary, ectopic expression of MBNL2 slightly reduced the phosphorylation levels of both kinases (Figure 4B). Next, we performed immunofluorescence staining to detect the phosphorylation level of Chk1 S345 in HCT116 cells and discovered that depletion of MBNL2 by shRNA increased the fluorescence intensity of Chk1 pS345 staining (Figure 4C). Furthermore, we examined the effect of MBNL2 on Chk1 phosphorylation in HeLa cells. Similarly, knockdown of MBNL2 enhanced the phosphorylation level of Chk1 S345, whereas overexpression showed weak effect (Figure 4D,E). 

### 2.5. MBNL2 Regulates DNA Damage Response Independently of p53 but Relying on p21

**Figure 5:**
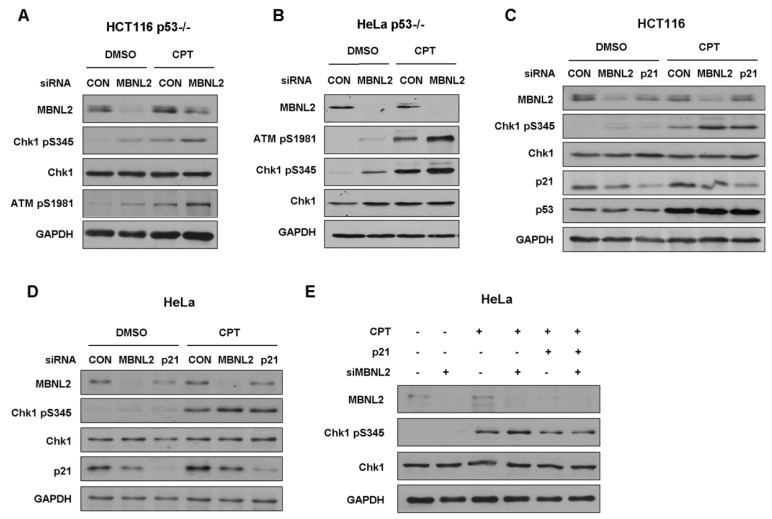
MBNL2 regulates the phosphorylation of Chk1 independently of p53 but through p21. (**A**) HCT116 p53−/− or (**B**) HeLa p53−/− cells transfected with control or MBNL2 siRNA were treated with 1 μM CPT for 4 h or mock-treated. The samples were subjected to immunoblotting analysis of Chk1 pS345 and ATM pS1981. (**C**) HCT116 or (**D**) HeLa cells were transfected with control, MBNL2, p21 siRNA, respectively, and treated with 1 μM CPT for 4 h or mock-treated. Samples were collected and subjected to immunoblotting analysis of Chk1 pS345. (**E**) HeLa cells were transfected with control or MBNL2 siRNA with/without p21-pGL3 plasmids and treated with 1 μM CPT for 4 h or mock-treated. The samples were subjected to immunoblotting analysis of Chk1 pS345.

Next, we investigated whether MBNL2 regulates DNA damage response in p53-null cells. Both HCT116 and HeLa p53−/− cells were transfected with control or MBNL2 siRNA and treated with CPT for 4 h to induce DNA damage response. Similar to the activation in wide type cells, depletion of MBNL2 significantly increased the phosphorylation levels of Chk1 S345 and ATM S1981 in both p53−/− cells (Figure 5A,B), indicating the regulatory effect of MBNL2 on DNA damage response is p53-independent.

Earlier studies demonstrated that p21 can regulate Chk1 activation both at transcriptional and post-translational levels [23,24]. Therefore, we examined whether MBNL2 regulates the activation of Chk1 through p21. We transfected control, MBNL2, or p21 siRNA in HCT116 or HeLa cells and treated cells with CPT for 4 h to induce DNA damage response. As expected, depletion of MBNL2 or p21 significantly increased the phosphorylation level of Chk1 S345 in both HCT116 and HeLa cells (Figure 5C,D). Moreover, concurrent overexpression of p21 rescued the increased Chk1 phosphorylation induced by MBNL2 knockdown, proving that MBNL2 regulates Chk1 activation and DNA damage response through p21 (Figure 5E).

### 2.6. MBNL2 Regulates Tumor Cell Fate after DNA Damage

**Figure 6:**
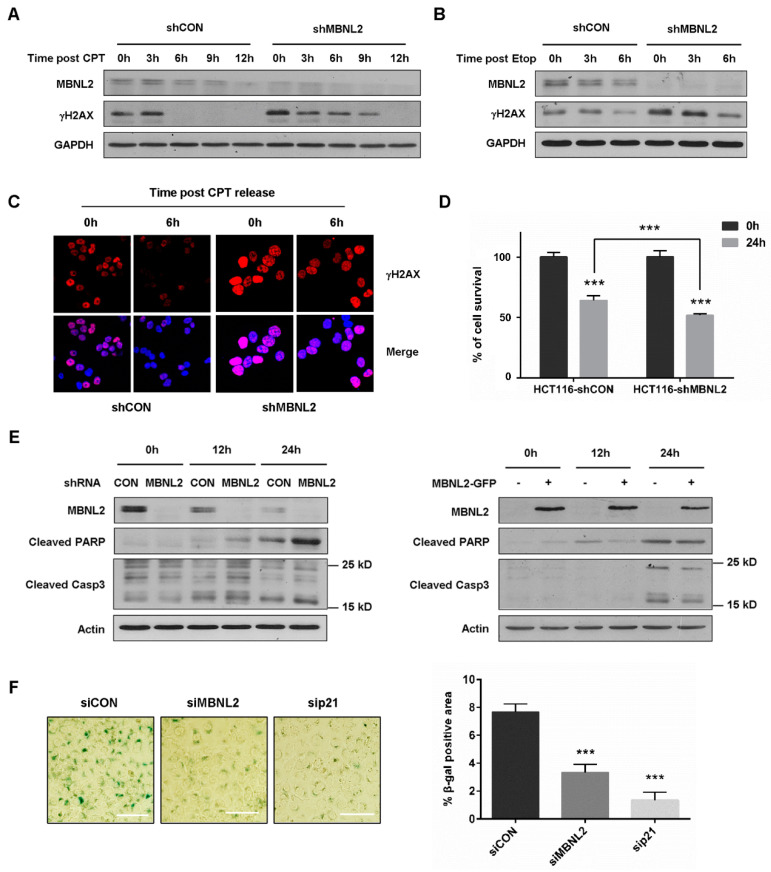
Depletion of MBNL2 impairs DNA damage repair and DNA damage-induced senescence but promotes DNA damage-induced apoptosis. (**A**) HCT116 cells transfected with control or MBNL2 shRNA lentiviral constructs were treated with 1 μM CPT, (**B**) 10 μM etoposide (Etop) for 12 h and released into a fresh medium for the indicated time. The samples were collected and subjected to immunoblotting analysis of γH2AX. (**C**) Representative images of γH2AX immunostaining in the HCT116 cells transfected with control or MBNL2 shRNA lentiviral constructs, treated with 1 μM CPT for 12 h, and released into a fresh medium for the indicated time. (**D**) HCT116 cells transfected with control or MBNL2 shRNA lentiviral constructs were treated with 1 μM CPT for 0 h or 24 h and the Cell Counting Kit-8 assay was performed to detect percentage of cell survival. (**E**) Immunoblotting of cleaved PARP and Caspase 3 in the HCT116 cells transfected with control or MBNL2 shRNA lentiviral constructs (left), empty vector, or MBNL2-GFP plasmids (right) and treated with 1 μM CPT for 0, 12, or 24 h. (**F**) Representative images (left) of SA-β-galactosidase staining in the HCT116 cells transfected with control, MBNL2, p21 siRNA and treated with 20 nM CPT for 96 h. The percentage of SA-β-galactosidase-positive cells was quantified (right). *** *p* < 0.001.

DNA damage response halts cell cycle progression to allow DNA damage repair, whereas severe DNA damage leads to apoptosis and cellular senescence. Since MBNL2 showed a regulatory effect on DNA damage response, we then investigated its impact on tumor cell fate upon DNA damage. HCT116 cells transfected with control or MBNL2 shRNA were treated with CPT for 12 h and then released from the drug to allow DNA damage repair. We used γH2AX, a sensitive marker of DNA double-strand breaks, to assess repair efficiency. γH2AX disappeared at 6 h after CPT release in control cells; however, it was still detected at 9 h after CPT release in MBNL2-depleted cells, revealing knockdown of MBNL2 significantly delayed DNA damage repair (Figure 6A). We also examined the effect of MBNL2 on repair of etoposide (Etop)-induced DNA damage. Similarly, depletion of MBNL2 delayed repair of Etop-induced DNA damage (Figure 6B). Immunofluorescent staining with the anti-γH2AX antibody in HCT116 cells showed that MBNL2 knockdown inhibited DNA damage repair as well, fluorescence in control cells declining much faster after CPT release than that in MBNL2-depleted cells (Figure 6C). 

Next, we investigated the effect of MBNL2 on DNA damage-induced apoptosis. MBNL2 depletion inhibited cell survival upon DNA damage (Figure 6D) and the hydrolytic cleavage of two significant markers of apoptosis, Caspase 3 and PARP were increased after MBNL2 knockdown and decreased upon MBNL2 overexpression (Figure 6E), indicating activation of DNA damage-induced apoptosis after MBNL2 depletion. DNA damage is a common driving factor of cellular senescence, so we lastly explored the effect of MBNL2 on DNA damage-induced senescence. HCT116 cells were transfected with control, MBNL2, or p21 siRNA and then treated with a low concentration of CPT for 96 h. Senescence-associated β-galactosidase (SA-β-galactosidase) has been widely used as a specific marker for senescent cells [25,26]. The SA-β-galactosidase assay showed that depletion of MBNL2 or p21 both strongly reduced the percentage of SA-β-galactosidase-positive cells, indicating an inhibitory effect of MBNL2 depletion on DNA damage-induced senescence (Figure 6F). Collectively, these results demonstrate that MBNL2 not only regulates DNA damage response, but also controls tumor cell fate after DNA damage.

## 3. Discussion

The cyclin-dependent kinase inhibitor p21 is a well-known negative regulator of cell cycle and a predominant transcriptional target of p53. p21 plays multiple functions in cell cycle arrest, DNA damage response, apoptosis, and cellular senescence [2]. The expression of p21 is tightly controlled at both transcriptional and post-transcriptional levels [2,27,28,29]. Many RNA-binding proteins have been reported to regulate p21 mRNA stability through binding to its 3′UTR [6,7,8,9,30,31,32]. In this research, we demonstrate that RNA-binding protein MBNL2 is a novel regulator of p21. The regulatory effect of MBNL2 on p21 is p53-independent; MBNL2 instead physically associates with p21 mRNA and modulates its stability. 

p21 is an active player in DNA damage response, and earlier studies have linked p21 induction to Chk1 inactivation. It is reported that p21 can transcriptionally repress Chk1 expression through the CDK2-pRb-E2F axis [24]. Deficient p21 induction or p21 knockdown is accompanied by increased/prolonged Chk1 activation, which could contribute to G2 arrest maintenance [23]. Here, we show that MBNL2 depletion downregulates p21 expression and induces Chk1 hyperphosphorylation upon DNA damage. And we further prove that MBNL2 regulates Chk1 activation and DNA damage response independent of p53 but relying on p21, concurrent overexpression of p21 rescuing the MBNL2 knockdown-induced Chk1 hyperphosphorylation. Noticeably, the effect of MBNL2 knockdown on p21 expression and Chk1 activation is far greater than that of MBNL2 overexpression. 

DNA damage response results in checkpoint activation and cell cycle arrest. To protect genome integrity, cells have evolved extensive signaling cascade to coordinate DNA damage repair with cell cycle progression. Upon severe DNA damage, however, cells irreversibly exit the cell cycle and enter senescence or undergo apoptosis [2]. There are several lines of evidence suggesting that p21 protects numerous cell types from genotoxic stress-induced apoptosis by inhibition of CDKs which are essential for activation of Caspase cascade [33,34]. On the other hand, persistent DNA damage response drives cellular senescence, and p21 contributes to the induction of cellular senescence through CDK inactivation and cell cycle arrest [35,36,37]. p21 maintains the viability of DNA damage-induced senescent cells, treatment with a low concentration of CPT inducing senescence in wild type cells, but apoptosis in p21-null cells [38,39]. In this study, we demonstrate that knockdown of MBNL2 delays DNA damage repair, promotes DNA damage-induces apoptosis, but inhibits cellular senescence. 

Multiple evidences support the existence of crosstalk between the PI3K/AKT pathway and the DNA damage response [40,41,42,43]. We performed transcriptome and gene expression analysis and found that the PI3K/AKT pathway is enriched in MBNL2-depleted cells. Except p21 and cyclin D1, several members of the PI3K/AKT pathway are significantly differentially expressed after MBNL2 depletion, hence, MBNL2 may regulate DNA damage response and tumor cell proliferation through other unknown mechanisms, which needs further investigation.

## 4. Materials and Methods 

### 4.1. Cell Culture and Treatment

HCT116 (purchased from ATCC, Manassas, VA, USA) and HCT116 p53−/− (provided by Prof. Zeping Hu, Tsinghua University), HeLa (purchased from ATCC) and HeLa p53−/− cells (purchased from EdiGene, Beijing, China) were maintained in RPMI 1640 and DMEM medium (Thermo Fisher Scientific, Waltham, MA, USA), respectively, both supplemented with 10% FBS (Biowest, France) and 1% Antibiotic–Antimycotic (Thermo Fisher Scientific, Waltham, MA, USA). For the operation of transient transfection, cells at a density of 2.0 × 10^5^ cells per well were transfected with 50 nM siRNA oligonucleotides (Santa Cruz Biotechnology, siMBNL2, sc-60990; sip21, sc-29427) or 1 μg/mL overexpression plasmids (synthetized from YouBio, Changsha, China) as indicated. All the transfections were performed in an opti-MEM medium (Thermo Fisher Scientific, Waltham, MA, USA), facilitated by Lipofectamine reagents (Thermo Fisher Scientific, Waltham, MA, USA) according to the manufacturer’s instructions. Six hours after transfection, the cells were transferred to a fresh medium. And 48 h after transfection, the cells were harvested and lysed for further experiments. To generate stable MBNL2-depleted and MBNL2-overexpressed cell lines, shMBNL2 and MBNL2-myc lentiviral constructs were purchased from Genechem and infected HCT116 cells. The lentivirus-infected cell lines were cultured under the selection pressure of 2 μg/mL puromycin (Thermo Fisher Scientific, Waltham, MA, USA). 

### 4.2. Transcriptome and Bioinformatics Analysis

Transcriptome analysis was performed by Gene Denovo Biotechnology Co. (Guangzhou, China). Briefly, total RNAs of control or MBNL2 siRNA-transfected HCT116 cells were extracted using the RNAiso Plus Reagent (TaKaRa, Mountain View, CA, USA). Then, mRNA was enriched by Oligo (dT) beads fragmented into short fragments and reverse-transcribed with random primers. The cDNA fragments were purified, end repaired, poly(A) added, and ligated to sequencing adapters. The ligation products were size-selected, PCR-amplified, and sequenced. Raw reads were filtered, mapped to the reference genome, reconstructed to transcripts, and annotated. The gene expression level was quantified and differently expressed genes were analyzed using edgeR (version 3.12.1) (http://www.r-project.org/). Genes with a fold change ≥ 2 and a false discovery rate (FDR) < 0.05 were considered significant differentially expressed genes, which were then subjected to enrichment analysis of GO functions and KEGG pathways [32]. 

### 4.3. Immunoblotting 

Cells were rinsed twice with PBS, collected, and lysed in a protein lysis buffer (5 mL 1 M Tris-HCl (pH 8.0), 26 g urea, 1 mL Triton X-100, 2 pieces of protease inhibitor cocktail, extended to the total volume of 100 mL with ddH_2_O) on ice for 30 min. The cell lysates were centrifuged at 12,000 rpm for 15 min at 4 °C and protein concentration was determined using the Bradford assay (Beyotime, Shanghai, China). The cell lysates were then supplemented with a loading buffer (8% SDS, 0.5% bromophenol blue, 40% glycerol, 8% β-mercaptoethanol) and boiled at 100 °C for 10 min. Proteins were separated using an SDS-PAGE gel and electrotransferred onto nitrocellulose membrane. Membrane was successively blocked in TBST with 5% milk, incubated with primary antibodies (anti-MBNL2, ab171551, 1:750; anti-Chk1 pS345, #2348, 1:1000; anti-Chk1, sc-8408, 1:500; anti-ATM pS1981, ab81292, 1:500; anti-GAPDH, abs118936, 1:20000; anti-p21, #2947, 1:1000; anti-γH2AX, #9718, 1:1000; anti-p53, 10442-1-AP, 1:8000; anti-cyclin D1, #29787, 1:500; anti-β-actin, 60008-1-Ig, 1:1000; anti-Caspase 3, #9662, 1:1000; anti-cleaved PARP, #9541, 1:1000) overnight at 4 °C, and then conjugated with KPL peroxidase-labeled goat anti-rabbit/mouse secondary antibodies (1:5000, 52200336, SeraCare, Milford, MA, USA) at room temperature for 1 h. Membrane was finally supplemented with the Pierce ECL Western Blotting Substrate (Thermo Fisher Scientific) and imaged with an autoradiography film (FUJIFILM, Minato, Japan). 

### 4.4. qRT–PCR

The cells were homogenized using 1 mL RNAiso Plus Reagent (TaKaRa, Mountain View, CA, USA), supplemented with 200 µL chloroform, and vibrated briefly. The mixture was then centrifuged (12,000 rpm, 4 °C, 15 min) into three layers. The clear top layer was carefully pipetted into a new tube. Equal volume of isopropanol was added, and total RNA was extracted after centrifugation (12,000 rpm, 4 °C, 10 min). The RNA pellet was then rinsed twice with 75% ethanol, dried, and dissolved in RNAase-free water. Complementary DNA was synthesized from 2 μg RNA using ReverTra Ace qPCR RT Master Mix (TOYOBO, Osaka, Japan) and qPCR was performed with SYBR Green Realtime PCR Master Mix (TOYOBO, Osaka, Japan) and primer pairs on a qTOWER 2.2 (Jena, Germany). The relative expression of target genes was normalized to GAPDH by the 2^−ΔΔCT^ method. Primer sequences are listed as follows: 

MBNL2, forward primer 5′-TCAAAGAGGAACATGCTCACG-3′, reverse primer 5′-AACGGCCCTTTAGGGAATCAA-3′;

p21, forward primer 5′-TGTCCGTCAGAACCCATGC-3′, reverse primer 5′-AAAGTCGAAGTTCCATCGCTC-3′;

cyclin D1, forward primer 5′-ACAAACAGATCATCCGCAAACAC-3′, reverse primer 5′-TGTTGGGGCTCCTCAGGTTC-3′;

GAPDH, forward primer 5′-AAGGCTGTGGGCAAGG-3′, reverse primer 5′-TGGAGGAGTGGGTGTCG-3′.

### 4.5. Immunofluorescence

The cells (2.0 × 10^5^) were plated onto a 20 mm circle cover glass and treated as indicated. The cells were first fixed with 4% formaldehyde for 10 min, and permeabilized with 0.25% Triton X-100 for 15 min. The cells were then successively blocked in PBS with 3% BSA for 1 h, probed with primary antibodies (anti-Chk1 pS345, #2348, 1:50; anti-γH2AX, 05-636, 1:250) overnight at 4 °C, and incubated with Alexa Fluor 488 or 555-conjugated secondary antibodies (1:400, Cell Signaling Technology, Danvers, MA, USA) at room temperature for 1 h. The cells were finally soaked in DAPI (Vector Laboratories, Burlingame, CA, USA), mounted on slides, sealed with nail polish, and dried in the dark. Images were acquired with a FLUOVIEW FV1000 (Olympus, Shinjuku, Japan) and quantified by ImageJ.

### 4.6. Flow Cytometry

The cells were first incubated with BrdU (1 mg/mL) for 0.5 h before harvesting. Next, the cells were rinsed twice with PBS, trypsized to single cells, and resuspended in a growth medium. Cell pellets were then fixed with 4 °C 70% ethanol/PBS for at least 30 min, permeabilized with 4N HCl at room temperature for 15 min, and resuspended in PBT (PBS + 0.5% BSA + 0.1% Tween 20). Finally, cell pellets were successively incubated in 200 µL PBT containing 1/40 dilution of anti-BrdU antibody (#5292, Cell Signaling Technology, Danvers, MA, USA) at room temperature for 30 min, 200 µL PBT containing 1/40 dilution of Alexa Fluor 488-conjugated secondary antibodies (Cell Signaling Technology, Danvers, MA, USA) at room temperature for 30 min, 1 mL PBS containing 250 µg/mL RNase A and 25 µg/mL PI at room temperature for 30 min. The percentage of different phases of cell cycle were analyzed (10,000 events for each sample) using a BD Accuri C6 flow cytometer (San Jose, CA, USA).

### 4.7. RNA-Binding Protein Immunoprecipitation (RIP)

The cells were transfected with empty vector or His-MBNL2 plasmids for 48 h and then the RIP assay was performed using a Magna RIP™ Kit (Millipore, Burlington, MA, USA) according to the manufacturer’s protocol. In brief, the cells were harvested in the RIP lysis buffer and incubated with rabbit IgG (ab172730) or anti-His-Tag (66005-1-Ig)-conjugated magnetic beads. RNAs bound by the immunoprecipitation magnetic complex were extracted and subjected to qRT-PCR analysis with the p21 primer.

### 4.8. Senescence-Associated β-Galactosidase (SA-β-Galactosidase) Assay

The cells were transfected with indicated siRNAs for 48 h and then treated with 20 nM CPT for 96 h. The SA-β-galactosidase assay was performed using an SA-β-galactosidase Staining Kit (Cell Signaling Technology, Danvers, MA, USA, #9860) according to the manufacturer’s protocol.

### 4.9. Colony Formation Assay

The cells (500–1000) were plated in 6 cm dishes and left for 8–10 days to allow colony formation. Then, colonies were rinsed with PBS, fixed with 4% formaldehyde for 15 min, and stained with 4% crystal violet for 30 min. Images were acquired and the number of colonies was counted with ImageJ.

### 4.10. Cell Counting Kit-8 (CCK-8) Assay

The cells (5 × 10^3^) were plated into 96-well plates with four repeats and treated as indicated. Then, the cells were transferred to a fresh medium supplemented with 10% CCK-8 solution for additional 2 h. After a gentle shake, OD_450_ values of each well were detected and analyzed by GraphPad Prism 7.0.

### 4.11. Statistical Analysis

The data were analyzed as the mean ± standard deviation (SD) of three independent experiments. All statistical analysis was performed using a two-tailed Student’s t-test. Statistical significance was depicted as follows: * *p* < 0.05; ** *p* < 0.01, *** *p* < 0.001.

## Data Availability

Data is contained within the article.

## Abbreviations

PI3K	Phosphatidylinositol 3-kinase
MBNL2	Muscleblind-like splicing regulator 2
CPT	Camptothecin
GAPDH	Glyceraldehyde 3-phosphate dehydrogenase
CDK	Cell cycle-dependent kinase
ATM	ataxia telangiectasia mutated
ATR	ataxia telangiectasia mutated and RAD3-related
Chk1	Checkpoint kinase 1
siRNA	Small interfering RNA
shRNA	Short hairpin RNA
RIP	RNA-binding protein immunoprecipitation
qRT–PCR	Quantitative reverse transcription PCR
KEGG	Kyoto Encyclopedia of Genes and Genomes
